# Identification of Potential Prognostic and Predictive Immunological Biomarkers in Patients with Stage I and Stage III Non-Small Cell Lung Cancer (NSCLC): A Prospective Exploratory Study

**DOI:** 10.3390/cancers13246259

**Published:** 2021-12-13

**Authors:** Rianne D. W. Vaes, Kobe Reynders, Jenny Sprooten, Kathleen T. Nevola, Kasper M. A. Rouschop, Marc Vooijs, Abhishek D. Garg, Maarten Lambrecht, Lizza E. L. Hendriks, Marijana Rucevic, Dirk De Ruysscher

**Affiliations:** 1 Department of Radiation Oncology (MAASTRO), GROW School for Oncology and Developmental Biology, Maastricht University Medical Center, 6200 MD Maastricht, The Netherlands; Kobe.Reynders@maastro.nl (K.R.); marc.vooijs@maastrichtuniversity.nl (M.V.); dirk.deruysscher@maastro.nl (D.D.R.); 2 Cell Stress & Immunity (CSI) Lab, Department for Cellular and Molecular Medicine, KU Leuven, 3000 Leuven, Belgium; jenny.sprooten@kuleuven.be (J.S.); abhishek.garg@kuleuven.be (A.D.G.); 3 OLINK Proteomics Inc., Waltham, MA 02453, USA; kathy.nevola@olink.com (K.T.N.); marijana.rucevic@olink.com (M.R.); 4 Department of Radiotherapy, GROW School for Oncology and Developmental Biology, Maastricht University Medical Center, 6200 MD Maastricht, The Netherlands; kasper.rouschop@maastrichtuniversity.nl; 5 Department of Radiotherapy-Oncology, Leuven Kanker Instituut, Universitair Ziekenhuis (UZ) Gasthuisberg, 3000 Leuven, Belgium; maarten.lambrecht@uzleuven.be; 6 Department of Pulmonary Diseases, GROW School for Oncology and Developmental Biology, Maastricht University Medical Center, 6200 MD Maastricht, The Netherlands; lizza.hendriks@mumc.nl

**Keywords:** non-small cell lung cancer, stereotactic body radiotherapy, chemoradiation, biomarkers, prognostic, predictive, immunogenic cell death

## Abstract

**Simple Summary:**

Over the last 5 years, immune checkpoint inhibitors (ICIs) are increasingly used in the treatment of non-small cell lung cancer (NSCLC) as either monotherapy or in combination with chemo- and/or radiotherapy. However, despite these advances, outcome still remains poor for most patients and there is still a lot of room to improve prognosis in these patients. To date, we have no tools that allow us to identify the patients that will benefit from chemo- and/or radiotherapy combined with immunotherapy, what treatment-induced immune changes can be expected, and what are the most optimal treatment combinations. Therefore, prognostic and predictive immunological biomarkers are urgently needed. This prospective exploratory study aimed to identify potential prognostic and predictive immune-related proteins that are associated with progression-free survival in patients with stage I/III NSCLC. The results of this trial provide a good starting point to implement blood-based immune profiling analyses in future clinical trials.

**Abstract:**

Radiotherapy (RT) and chemotherapy can induce immune responses, but not much is known regarding treatment-induced immune changes in patients. This exploratory study aimed to identify potential prognostic and predictive immune-related proteins associated with progression-free survival (PFS) in patients with non-small cell lung cancer (NSCLC). In this prospective study, patients with stage I NSCLC treated with stereotactic body radiation therapy (*n* = 26) and patients with stage III NSCLC treated with concurrent chemoradiotherapy (*n* = 18) were included. Blood samples were collected before (v1), during (v2), and after RT (v3). In patients with stage I NSCLC, CD244 (HR: 10.2, 95% CI: 1.8–57.4) was identified as a negative prognostic biomarker. In patients with stage III NSCLC, CR2 and IFNGR2 were identified as positive prognostic biomarkers (CR2, HR: 0.00, 95% CI: 0.00–0.12; IFNGR2, HR: 0.04, 95% CI: 0.00–0.46). In addition, analysis of the treatment-induced changes of circulating protein levels over time (Δv2/v3−v1) also identified CXCL10 and IL-10 as negative predictive biomarkers (CXCL10, HR: 3.86, 95% CI: 1.0–14.7; IL-10, HR: 16.92 (2.74–104.36)), although serum-induced interferon (IFN) response was a positive prognostic. In conclusion, we identified several circulating immunogenic proteins that are correlated with PFS in patients with stage I and stage III NSCLC before and during treatment.

## 1. Introduction

Over the last 5 years, developments in the treatment of non-small cell lung cancer (NSCLC) have increasingly focused on the efficacy of immunotherapy as either monotherapy or in combination with chemotherapy, other immunotherapy, and/or radiotherapy [1]. Immune checkpoint inhibitors (ICIs) such as nivolumab and pembrolizumab, both monoclonal antibodies that target programmed death 1 (PD-1), are widely used in the metastatic setting [2,3,4,5,6,7,8]. Recent updated results from the randomized, placebo-controlled, phase III PACIFIC trial revealed a significantly improved overall survival (OS) and progression-free survival (PFS) at 5 years with the addition of the ICI durvalumab (anti-PD-L1) vs. placebo, given for 12 months after concurrent chemoradiation (CCRT) [9,10,11,12,13]. After 5 years, 43% of patients randomized to durvalumab remained alive and nearly one third of patients treated with durvalumab remained alive without disease progression [13]. As a result, adjuvant durvalumab has become standard of care for patients with locally advanced (stage III) NSCLC treated with CCRT.

However, even with the introduction of immunotherapy in the curative treatment setting, outcome remains poor and improving the prognosis of patients with NSCLC continues to be difficult as most patients either are unresponsive or experience rapid tumor progression after an initial response. Therefore, biomarkers that can aid in the selection of patients that will most likely benefit from immunotherapy or patients that develop resistance to ICIs during treatment are urgently needed. In current clinical practice, the level of PD-L1 on the tumor cell surface (assessed by immunohistochemistry) is an approved biomarker to select patients with NSCLC that are eligible to be treated with ICIs [14,15]. However, it remains challenging to find a clear predictive PD-L1 threshold; less than half of biomarker-selected patients benefit from treatment and some clinical responses may be encountered in “PD-L1-negative” cohorts [16,17]. Simultaneously, the clinical benefit of ICIs stands or falls with the strength of the elicited anti-tumor immune response. Given that the immune response in untreated and treated patients with cancer involves both immunostimulatory and immunosuppressive processes, it is not likely that the clinical response to ICIs can be captured by one single biomarker.

As ICIs predominantly lift the brakes of the immune system, it is logical to combine ICIs with other treatment modalities that can induce strong anti-tumor immune responses. To date, it has been well-established that radiotherapy (RT) can induce tumor-targeting immune responses, which critically rely on the antigenicity of cancer cells and their capacity to generate adjuvant signals [18,19]. In particular, RT can induce an immunogenic variant of regulated cell death, called immunogenic cell death (ICD), that results in the expression and/or release of numerous danger-associated molecular patterns (DAMPs) and cytokines [20], including ATP [21,22], cellular nucleic acids [23,24,25], high mobility group box 1 (HMGB1) [26], ANXA1 [27], cytokines such as interferon type I (IFN-I), CCL2, CXCL1, and CXCL10 [24,28,29], and ER chaperones such as calreticulin, ERp57, Hsp70, and Hsp90 [30,31,32]. These ICD-associated molecules are exposed or released by dying tumor cells and are sensed by innate immune cells, hence they can promote the activation and maturation of these cells in order to elicit an adaptive immune response.

In the clinical setting, we have no tools to identify patients that will benefit from RT treatment combined with immunotherapy, or patients that exhibit primary or secondary (acquired) resistance to immunotherapy [33]. Therefore, profiling the extent of RT-induced immunogenic responses in individual patients with NSCLC will be an essential first step. Emerging evidence suggests that stereotactic body radiotherapy (SBRT), an RT modality that delivers very high doses of RT to the cancer cells, results in excellent local tumor control and has a higher immunomodulatory potential compared to conventional radiotherapy [34,35]. Despite intensive research on the immunomodulatory properties of RT, the potential RT-induced immune changes in patients with NSCLC remain largely unknown.

In this prospective exploratory study, we aimed to identify potential prognostic and predictive immune-related proteins that are associated with PFS in patients with stage I and stage III NSCLC treated with RT. First, we set out to determine whether circulating levels of ICD-related proteins (i.e., HMGB1, ANXA1, CALR, and CXCL10) at baseline and during RT treatment were associated with PFS. Secondly, we used a targeted proteomic-based approach to enable a broader screening of the plasma proteome and the association thereof with PFS. Here, we report the identification of several circulating immunogenic proteins that are correlated with PFS in patients with stage I and stage III before and/or during treatment.

## 2. Materials and Methods

### 2.1. Patients

Patients diagnosed with stage I or stage III NSCLC scheduled for curative intent RT were prospectively included at MAASTRO (Maastricht, The Netherlands) and University Hospitals Leuven (UZ Leuven, Leuven, Belgium) between May 2017 and March 2019. Patients were treated according to standard of care treatment programs. Patients with stage II and stage IV NSCLC were excluded as the majority of these patients are undergoing surgery only or receiving palliative treatment, respectively. Patients with stage I NSCLC (group 1) were treated with SBRT (54 Gy in 3 fractions, 48 Gy in 4 fractions, or 60 Gy in 8 fractions). Patients with stage III NSCLC (group 2) were treated with concurrent platinum-doublet chemotherapy (cisplatin or carboplatin combined with etoposide) and radiotherapy (60–66 Gy in 2 Gy fractions, five times a week). Blood was sampled immediately before the first fraction of RT, immediately before the second (group 1) or third (group 2) fraction of RT, and immediately after the last fraction of RT. Key exclusion criteria include the chronic use of corticosteroids (except when used as anti-emetics for chemotherapy or inhalers); the use of NSAIDs taken until 5 days before RT or during RT; active auto-immune diseases; and the use of immunosuppressive medication. All patients provided written informed consent. This study was approved by the local Medical Ethics Committee (NL59321.068.16/METC 163073) and was registered in ClinicalTrials.gov Identifier: (NCT02921854).

### 2.2. Clinical Data Collection

At baseline, the following patients’ characteristics were collected: gender, age, body weight, and tumor type. OS was calculated to the time of death due to any cause. PFS was determined by calculating the time from receiving the first fraction of RT to the time of progressive disease. Data cut-off date was per 22 February 2021.

### 2.3. Blood Collection

At the indicated time-points, blood was collected in EDTA tubes and stored on ice until further processing. The blood was centrifuged at 1580× *g* at 4 °C for 15 min without brake. Plasma aliquots were stored at −80 °C until further analysis.

### 2.4. Proximity Extension Assay (Olink Proteomics)

The abundance of >1000 proteins were analyzed in the collected plasma samples using the Olink^®^ Target 96 panels (cardiometabolic, cardiovascular II, cardiovascular III, cell regulation, development, immune response, inflammation, neurology, oncology II, oncology III, and organ damage) (Olink Proteomics, Uppsala, Sweden). In each panel, protein abundance was measured by the Proximity Extension Assay (PEA) technology (Olink Proteomics, Uppsala, Sweden). The PEA is an affinity-based assay which characterizes abundance levels of a pre-determined set of proteins. For each measured protein, a pair of oligonucleotide-labeled antibody probes target the protein and if both probes are in close proximity, a PCR target sequence is formed by a proximity-dependent DNA polymerization event. The resulting sequence is then detected and quantified using standard real-time PCR. The protein abundance is reported as Normalized Protein eXpression (NPX), Olink’s arbitrary relative unit, which is in Log2 scale. The NPX data are used to identify differences at baseline and changes during treatment for individual proteins in blood samples from both patients with stage I and stage III NSCLC.

### 2.5. Enzyme-Linked Immunosorbent Assay (ELISA)

HMGB1 protein was determined in patient plasma (100 μL; 1:1 diluted in PBS) using HMGB1 ELISAs (Novus Biologicals, Cat nr. NBP2-62766, Abingdon, UK), according to the manufacturers’ instructions.

### 2.6. Serum-Functional Immunodynamic Status (sFIS) Assay

This sFIS assay [36] consisted of THP1-Dual™ myeloid reporter cells (InvivoGen, Toulouse, France) seeded in a 96-well plate at a density of 30,000 cells per well in 100 µL media (RPMI-1640 containing 2 mM L-glutamine, 25 mM HEPES, 100 units/mL penicillin, 100 µg/L streptomycin, 100 µg/mL Normocin and 10% heat inactivated fetal calf serum). After 24 h, THP1 cells were treated with 100 µL of patient plasma for 24 h. To determine activation of interferon-stimulated response elements (ISRE) coding for interferon (IFN)-stimulated genes (ISG), luciferase was checked in media by adding 50 µL of Quanti-Luc GOLD (InvivoGen) to 100 µL of (separately recovered) THP1 media. Bioluminescence was examined for 100 ms immediately after Quanti-Luc GOLD addition by microplate reader (Agilent, Santa Clara, CA, USA). IFN/ISG response activity values were normalized using the results of samples taken before RT treatment of each individual patient.

### 2.7. Statistical Analysis

Patient data and outcome parameters were entered in GraphPad Prism (version 9.1.2) and analyzed using the non-parametric Mann–Whitney U test (continuous variables) to compare differences between two groups. To compare differences between more than 2 groups, the non-parametric Kruskal–Wallis test was applied followed by Dunn’s post-hoc testing with Bonferroni correction. The Fisher’s exact test was used for between-group comparisons of categorical variables. Protein abundance data were analyzed using the Cox Proportional-Hazards Model and Benjamini–Hochberg multiple hypothesis correction for patients with stage I and stage III NSCLC (“R”, version 4.0.3). One sample was determined to be an outlier by PCA plots and by sample median vs. sample IQR plots. This sample was excluded from stage I analysis. Samples with a QC-warning status were also excluded on a per-panel basis from both analyses. Results were then adjusted for multiple testing using the Benjamini–Hochberg method. Hazard Ratio is based on a 1 NPX difference. The association between baseline NPX (v1) and delta NPX (Δv2/v3−v1) plasma protein levels and PFS of the patients were assessed by plotting Kaplan–Meier curves and logrank *p* values were calculated. Patients with stage I and stage III NSCLC were assessed separately. Patients were stratified into low or high expression-based “risk groups” by considering the median expression value of each parameter as a cut-off. For all statistical analyses, a nominal *p*-value less than 0.05 was considered to be statistically significant.

## 3. Results

### 3.1. Patient Characteristics

**Figure 1:**
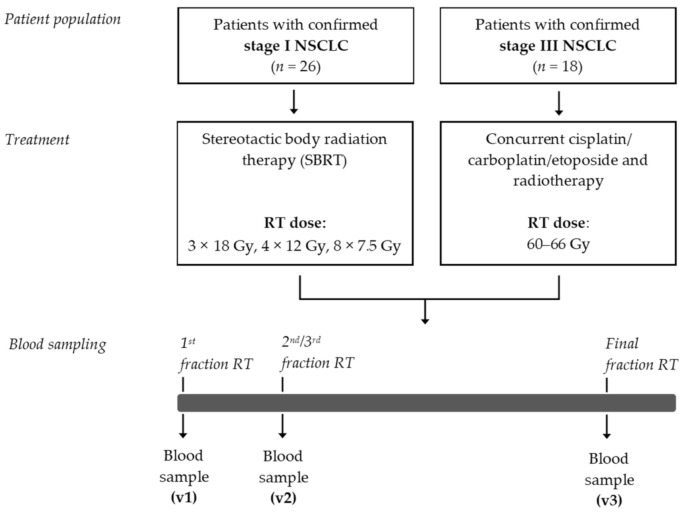
Study design. NSCLC, non-small cell lung cancer; RT, radiotherapy; SBRT, stereotactic body radiotherapy.

A flow chart of this exploratory study is depicted in Figure 1. Between May 2017 and March 2019, a total of 44 patients were enrolled in this study. The patient characteristics are presented in Table 1. Mean age of this patient population was 68.6 (±9.7) years, and 61.4% were male. The age and gender distribution among these patients are comparable to the age and gender distribution that has recently been reported in a nationwide population-based study in The Netherlands [37]. Of these 44 patients, 26 patients were diagnosed with stage I NSCLC. These patients were not eligible to undergo surgical resection of the primary tumor and were therefore treated with curative-intent SBRT. The median PFS was 25.0 (±11.5) months. Eighteen patients were diagnosed with stage III NSCLC and were treated with CCRT. The median PFS was 7.8 (±12.8) months.

### 3.2. Detection of Immunogenic Cell Death-Related Proteins in the Circulation of Patients with Stage I NSCLC Treated with SBRT

**Figure 2:**
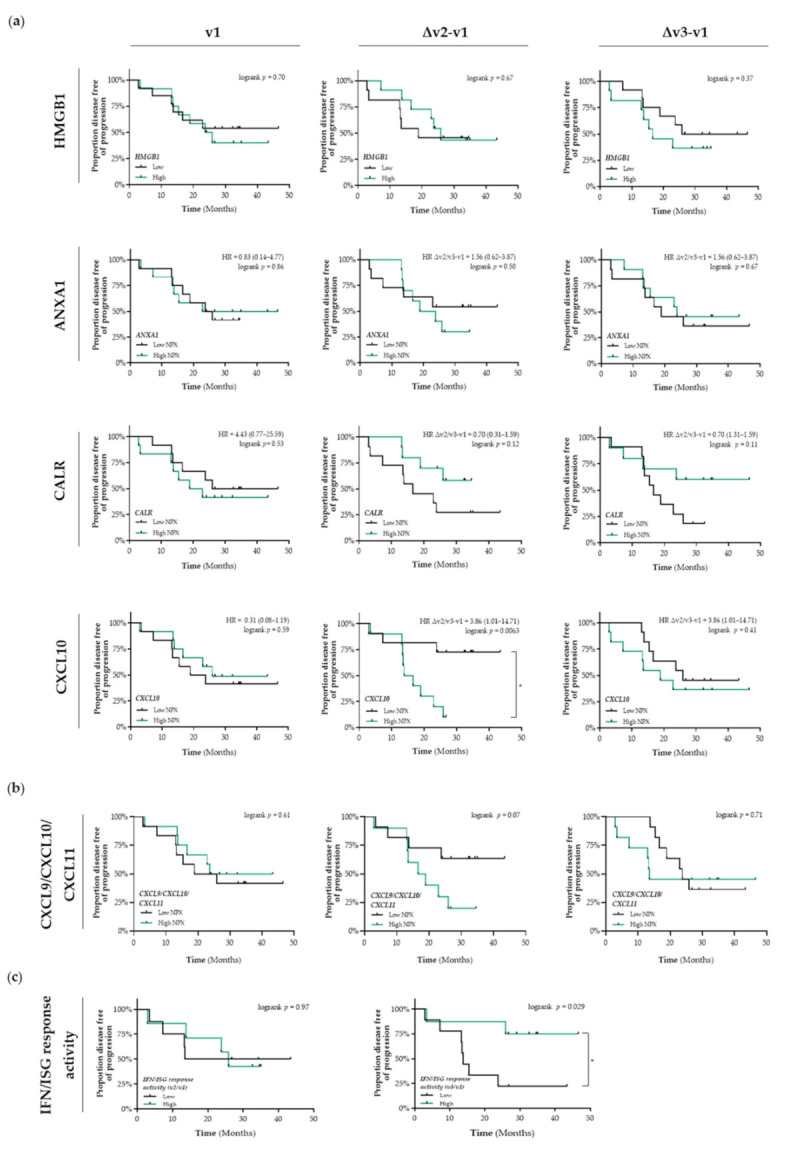
Detection of immunogenic cell death-related proteins in the circulation of patients with stage I NSCLC treated with SBRT. (**a**) Circulating levels of immunogenic cell death (ICD)-related proteins in patients with stage I NSCLC (*n* = 25) at baseline (v1), after the 2nd fraction of SBRT (v2), after the final fraction of SBRT (v3) were assessed by ELISA (HMGB1) or by the Proximity Extension Assay from Olink (ANXA1, CALR, and CXCL10). Patients were stratified into low (black lines) or high (green lines) expression-based “risk groups” by considering the median expression value of each protein as a cut-off, followed by Kaplan–Meier plotting on the patient’s progression free survival (PFS). (**b**) The CXCL9/CXCL10/CXCL11 chemokine profile was generated by calculating the mean NPX. Patients were stratified into low (black lines) or high (green lines) NPX-based “risk groups” by considering the median NPX value of the mean NPX (CXCL9, CXCL10, and CXCL11) as a cut-off. (**c**) Plasma-stimulated activation of interferon-stimulated response elements (ISRE) coding for interferon (IFN)-stimulated genes (ISG) was assessed by measuring luciferse activity in media from THP1-Dual™ reporter cells that were treated for 24 h with plasma samples. IFN/ISG response activity values were normalized against the IFN/ISG activity measured in media from THP1-Dual™ reporter cells treated for 24 h with pre-treatment (v1) samples of each individual patient. Patients were stratified into low (black lines) or high (green lines) IFF/ISG response activity-based “risk groups” by considering the median IFN/ISG activity value as a cut-off. In all graphs, respective logrank test *p*-values and Hazard Ratios (HR with its 95% confidence interval in parenthesis) are displayed. A nominal *p*-value less than 0.05 was considered to be statistically significant and is indicated through an asterisk (*).

In untreated patients with stage I NSCLC (v1), the differential circulating levels (Figure S1a) of HMGB1, ANXA1, CALR, and CXCL10 were not associated with PFS (Figure 2a). Patients that were treated with SBRT and having increased circulating levels of CALR tended to have a better PFS than patients with low circulating levels of CALR (Δv2/v3−v1; HR = 0.7, 95% CI: 0.31–1.59; Δv2−v1 logrank *p* = 0.12, Δv3−v1 logrank *p* = 0.11) (Figure 2a). Shortly after the first fraction of RT, increased circulating levels of CXCL10 were significantly associated with a shorter PFS compared to patients with low circulating levels of CXCL10 (HR = 3.86, 95% CI: 1.01–14.7; logrank Δv2−v1 *p* = 0.0063). Similar as for CXCL10 alone, we observed that increased circulating levels of the CXCL9/CXCL10/CXCL11 chemokine profile (known to be predominantly induced by IFN-γ) tended to be associated with a lower PFS (Δv2−v1 logrank *p* = 0.07) (Figure 2b). Finally, increased plasma-induced myeloid IFN/ISG response activity at the end of SBRT treatment was significantly associated with a better PFS (logrank *v3/v1 p* = 0.029) (Figure 2c).

### 3.3. Detection of Immunogenic Cell Death-Related Proteins in the Circulation of Patients with Stage III NSCLC Treated with CCRT

**Figure 3:**
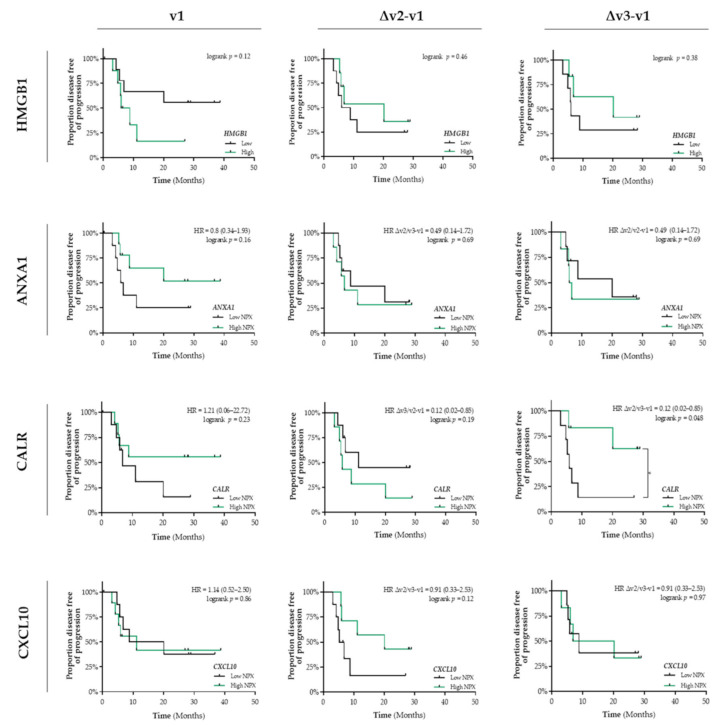
Detection of immunogenic cell death-related proteins in the circulation of patients with stage III NSCLC treated with CCRT. Circulating levels of immunogenic cell death (ICD)-related proteins in patients with stage III NSCLC (*n* = 18) at baseline (v1), after the 3rd fraction of RT (v2), after the final fraction of RT (v3) were assessed by ELISA (HMGB1) or by the Proximity Extension Assay from Olink (ANXA1, CALR, and CXCL10). Patients were stratified into low (black lines) or high (green lines) expression-based “risk groups” by considering the median expression value of each protein as a cut-off, followed by Kaplan–Meier plotting on the patient’s progression free survival (PFS). In all graphs, respective logrank test *p*-values and Hazard Ratios (HR with its 95% confidence interval in parenthesis) are displayed. A nominal *p*-value less than 0.05 was considered to be statistically significant and is indicated through an asterisk (*).

In addition, in untreated patients (v1) with stage III NSCLC, the differential circulating levels (Figure S1b) of HMGB1, ANXA1, CALR, and CXCL10 were not associated with PFS (Figure 3). Only a similar predictive expression pattern was observed for circulating CALR levels. Increased levels of CALR at the end of CCRT treatment is significantly associated with a better PFS (HR = 0.12, 95% CI: 0.02–0.85; logrank Δv3−v1 *p* = 0.048) (Figure 3). In contrast to our observations in patients with stage I NSCLC, we did not observe any association between the interferon-inducible chemokines (CXCL9/CXCL10/CXCL11) or IFN/ISG response activity and PFS (Figure S2).

### 3.4. Identification of Potential Prognostic Immune-Related Proteins in Patients with NSCLC

**Figure 4:**
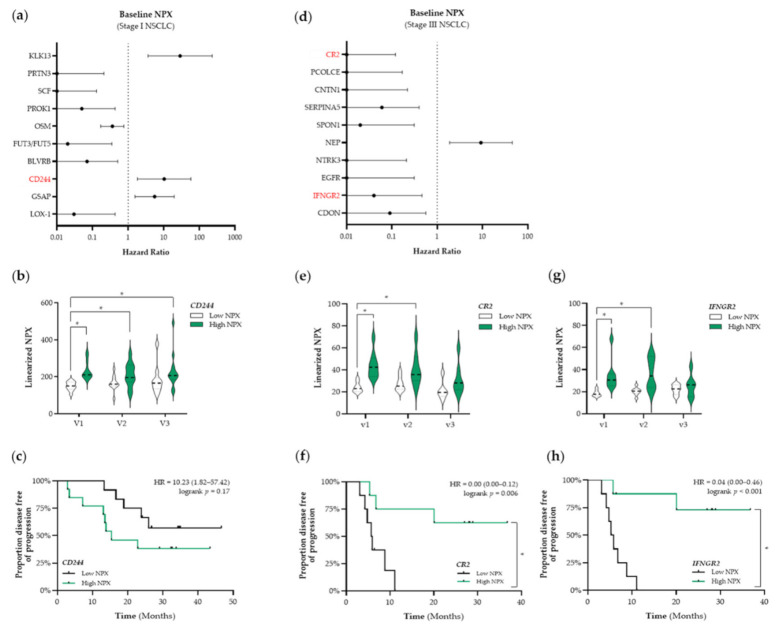
Identification of prognostic immune-related proteins in patients with NSCLC. (**a**) The Cox Proportional-Hazards model was used to identify proteins associated with PFS at baseline (v1) in patients with stage I NSCLC (*n* = 25). The top 10 proteins associated with PFS in patients with stage I are displayed in a forest plot. The closed circles represent the Hazard Ratio of each identified protein and the horizontal lines illustrate the 95% confidence interval. Immune-related proteins are indicated in red. (**b**,**c**) Patients with stage I NSCLC (*n* = 25) were stratified into low (black) or high (green) CD244 (Signaling Lymphocyte Activation Molecule (SLAM) family immunoregulatory receptor) NPX-based “risk groups” by considering the median NPX value of the CD244 protein at v1 as a cut-off. (**b)** The linearized CD244 NPX values are presented in a violin plot, showing the distribution of the CD244 NPX values at each timepoint. The median is indicated by the dashed line, the quartiles are indicated by the dotted lines. (**c**) Kaplan–Meier plot of the low CD244 NPX group and high CD244 NPX group on the patients’ PFS. (**d**) The Cox Proportional-Hazards model was used to identify proteins associated with PFS at baseline (v1) in patients with stage III NSCLC (*n* = 18). The top 10 proteins associated with PFS in patients with stage III are displayed in a forest plot. Immune-related proteins are indicated in red. (**e–h**) Patients with stage III NSCLC (*n* = 18) were stratified into low (black) or high (green) NPX-based “risk groups” by considering the median NPX value of the protein at v1 as a cut-off. (**e**) The linearized CR2 (complement receptor 2) NPX values are presented in a violin plot, showing the distribution of the CR2 NPX values at each timepoint. (**f**) Kaplan–Meier plot of the low CR2 NPX group and high CR2 NPX group on the patient’s PFS. (**g**) The linearized IFNGR2 NPX values are presented in a violin plot, showing the distribution of the IFNGR2 NPX values at each timepoint. (**h**) Kaplan–Meier plot of the low IFNGR2 NPX group and high IFNGR2 NPX group on the patient’s PFS. In all Kaplan–Meier plots, respective logrank test *p*-values and Hazard Ratios (HR with its 95% confidence interval in parenthesis) are displayed. To compare differences between groups, the non-parametric Kruskal–Wallis test was applied followed by Dunn’s post-hoc testing with Bonferroni correction. A nominal *p*-value less than 0.05 was considered to be statistically significant and is indicated through an asterisk (*).

Based on these results, we decided to use a targeted proteomic-based approach to enable a broader screening of the plasma proteome and the association thereof with PFS. First, we aimed to identify prognostic factors that were associated with PFS. The top 10 identified proteins that are associated with PFS in patients with stage I and stage III NSCLC are presented in Figure 4a,d, respectively. Given our initial focus on the potential ICD-inducing properties of SBRT and CCRT, we only selected immune-related proteins for further analyses (shown in red in Figure 4). In patients with stage I NSCLC, we identified CD244 as a potential prognostic protein (HR: 10.23, 95% CI: 1.82–57.42) (Figure 4a). Stratification of patients with stage I NSCLC into low and high NPX-based “risk groups” by considering the median NPX value of CD244 at baseline as a cut-off, revealed significant differences between both “risk groups”. Compared to the low CD244 NPX-based “risk group” at baseline, CD244 NPX values were significantly higher in the high NPX-based “risk group” at baseline (v1 “low CD244” vs. v1 “high CD244”, *p* < 0.001), after the second fraction of SBRT (v1 “low CD244” vs. v2 “high CD244”, *p* = 0.029), and after the final fraction of SBRT (v1 “low CD244” vs. v3 “high CD244”, *p* = 0.0056) (Figure 4b). Survival analysis revealed that the high CD244 NPX-based “risk group” tended to be associated with a worse PFS (logrank *p* = 0.17) (Figure 4c).

In patients with stage III NSCLC, we identified complement receptor type 2 (CR2) and interferon gamma receptor 2 (IFNGR2) as potential prognostic proteins (CR2–HR: 0.00, 95% CI: 0.00–0.12; IFNGR2–HR: 0.04, 95% CI: 0.00–0.46) (Figure 4d). Stratification of patients with stage III NSCLC into low and high NPX-based “risk groups” by considering the median NPX value of either CR2 or IFNGR2 at baseline as a cut-off also revealed significant differences between both groups. CR2 NPX values in the high NPX-based “risk group” at baseline (v1) and after the third fraction of RT (v2) were significantly higher compared to the NPX values in the CR2 low-based “risk group” at baseline (v1 “low CR2” vs. v1 “high CR2”, *p*= 0.007; v1 “low CR2” vs. v2 “high CR2”, *p* = 0.045, respectively) (Figure 4e). Survival analysis revealed that increased plasma levels of CR2 at baseline are significantly associated with a better PFS (logrank *p* = 0.006, Figure 4f). IFNGR2 NPX values in the high NPX-based “risk group” at baseline (v1) and after the third fraction of RT (v2) were significantly higher compared to the NPX values in the IFNGR2 low-based “risk group” at baseline (v1 “low IFNGR2” vs. v1 “high IFNGR2”, *p* = 0.0022; v1 “low IFNGR2” vs. v2 “high IFNGR2”, *p* = 0.01) (Figure 4g). Survival analysis revealed increased plasma levels of IFNGR2 at baseline is significantly associated with a better PFS as well (logrank, *p* < 0.001, Figure 4h).

### 3.5. Identification of Potential Predictive Immune-Related Proteins in Patients with NSCLC

**Figure 5:**
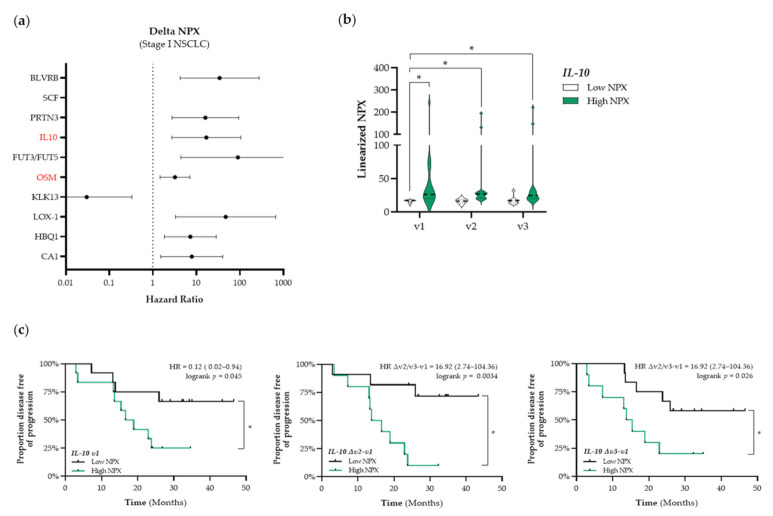
Identification of predictive immune-related proteins that are altered in response to therapy in patients with stage I NSCLC. (**a**) The Cox Proportional-Hazards model was used to identify proteins of which the delta NPX value was associated with PFS in patients with stage I NSCLC (*n* = 25). The top 10 proteins of which the delta NPX value was associated with PFS in patients with stage I are displayed in a forest plot. The closed circles represent the Hazard Ratio of each identified protein and the horizontal lines illustrate the 95% confidence interval. Immune-related proteins are indicated in red. (**b**,**c**) Patients with stage I NSCLC (*n* = 25) were stratified into low (black) or high (green) IL-10 (interleukin-10) NPX-based “risk groups” by considering the median NPX value of the IL-10 protein at v1 as a cut-off. (**b**) The linearized IL-10 NPX values are presented in a violin plot, showing the distribution of the IL-10 NPX values at each timepoint. The median is indicated by the dashed line, the quartiles are indicated by the dotted lines. (**c**) Kaplan–Meier plots of the low IL-10 NPX group and high IL-10 NPX group on the patient’s PFS at baseline (v1) (**left***)*, after the 2nd fraction of SBRT (Δv2−v1) (**middle**), and after the final fraction of SBRT (Δv3−v1) *(***right**). A nominal *p*-value less than 0.05 was considered to be statistically significant and is indicated through an asterisk (*).

In addition to the identification of potential prognostic immune-related proteins, we investigated whether treatment-induced changes in the expression levels of plasma proteins over time were associated with outcome. The top 10 identified proteins that are associated with PFS in patients with stage I NSCLC are presented in Figure 5a. Again, we only selected immune-related proteins for further analyses (shown in red in Figure 5a). We identified interleukin-10 (IL-10) and Oncostatin-M (OSM) as potential predictive proteins (HMGB1, ANXA1, CALR, and CXCL10) before the start of RT treatment (SBRT and CCRT, respectively) that were not associated with PFS. During RT treatment, increased circulating levels of CALR were associated with a longer PFS and increased circulating levels of CXCL10 were associated with a shorter PFS. In stressed or dying cells, CALR exposed on the cell surface serves as a phagocytic signal to dendritic cells (DCs), resulting in the engulfment of cancer cells by DCs, tumor antigen presentation, and anti-cancer cytotoxic T lymphocyte-specific immune responses [31]. Numerous preclinical studies have already shown the potential of radio- and chemotherapy to induce upregulation of CALR on the proteins (IL-10, HR: 16.92, 95% CI: 2.74–104.36; OSM, HR: 3.22, 95% CI: 1.46–7.08). As the circulating OSM expression levels were correlated with the COPD status of these patients, OSM was not further considered as a potential predictive biomarker due to bias (Figure S3). By stratifying patients with stage I NSCLC into low and high NPX-based “risk groups” by considering the median NPX value of IL-10 at baseline as a cut-off we observed that differences in IL-10 expression were already present at baseline and seemed not to be affected by SBRT (Figure 5b). IL-10 NPX values in the high NPX-based “risk group” at baseline (v1), after the second fraction of SBRT (v2), and after the final fraction of SBRT (v3) were significantly higher compared to the NPX values in the IL-10 low-based “risk group” at baseline (v1 “low IL-10” vs. v1 “high IL-10”, *p* < 0.001; v1 “low IL-10” vs. v2 “high IL-10”, *p* < 0.001; v1 “low IL-10” vs. v3 “high IL-10”, *p* = 0.0019). In addition, Kaplan–Meier survival analysis revealed that high plasma IL-10 levels are significantly associated with a worse PFS at baseline (logrank v1, *p* = 0.045), during SBRT (logrank Δv2−v1, *p* = 0.003), and at the end of treatment (logrank Δv3−v1, *p* = 0.03) (Figure 5c). In patients with stage III NSCLC, no important changes in immune-related proteins were found.

## 4. Discussion

Biomarkers that can aid in the selection of patients that will most likely benefit from immunotherapy or patients that develop resistance to ICIs are currently lacking in clinical practice. The principal goal of this prospective exploratory study was therefore to identify potential predictive and prognostic immune-related proteins in patients with NSCLC. Here, we have identified several immunogenic-related proteins that show prognostic merit. The expression levels of circulating immunogenic proteins remained largely unaltered during treatment.

In patients with stage I and stage III NSCLC, the circulating levels of key ICD-related proteins before the start of RT treatment were not associated with PFS. During RT, increased circulating levels of CALR and CXCL10 were associated with either a longer or shorter PFS, respectively. Numerous preclinical studies have already shown that radio- and chemotherapy can result in higher expression levels of CALR on the tumor cell surface, resulting in enhanced anti-tumor immune responses [38,39,40,41]. In addition, Garg et al. previously showed that patients with NSCLC, treated with RT and having high CALR expression levels in the tumor, tended to have significantly better OS compared with RT-treated NSCLC patients with low CALR expression [42]. Here, we showed that the treatment-induced upregulation of circulating CALR levels is associated with a better PFS. However, given the location and function of CALR on stressed or dying cells, it remains questionable to what extent circulating CALR levels reflect the treatment-induced upregulation of CALR on the surface of tumor cells. Increasing evidence suggests that CALR can be released by cancer cells and may serve as a predictive biomarker. In an in vitro study, Garg et al. showed that ROS-induced ER stress in cancer cells resulted in the exposure of CALR on the tumor cell surface, followed by the passive extracellular release of CALR [43]. In addition, in line with our results, Inoue et al. recently showed that an overall increase of circulating CALR levels in patients with advanced lung cancer treated with platinum-based combination or single-agent chemotherapy tended to be associated with a clinical tumor response [44]. Thus, released and/or circulating levels of CALR may reflect the local treatment-induced changes of CALR expression on the tumor cell level.

CXCL10 is a chemotactic factor that belongs, together with CXCL9 and CXCL11, to the IFN-γ inducible chemokines of the CXC-chemokine family that all share CXCR3 as a common receptor for their immune-activating activities. Treatment-driven ICD involves the activation of a cancer cell-intrinsic type I IFN response and consequent secretion of CXCL10 that mediates chemotactic effects of activating immune cells, including cytotoxic CD8+ T-cells and NK-cells, into the tumor microenvironment [19,45]. Given that CXCL10 expression by tumors is associated with effective anti-tumor immunity and CD8+ T-cell regulation [46], we did not expect to observe a negative correlation between circulating levels of CXCL10 and PFS in patients with stage I NSCLC treated with SBRT. However, in line with our observations, previous studies have also shown similar results in patients with other cancer types; high levels of CXCL10 were correlated with tumor recurrence or even a lower survival [47,48,49]. As such, the association between CXCL10 and a poor outcome may be the consequence of the pro-tumorigenic properties of CXCL10. In multiple cancer models, it has been shown that CXCL10 induces cancer cell proliferation and upregulates invasion-related properties [47,49,50,51]. Altogether, the dual effects of CXCL10 on the tumor and immune cells may occur simultaneously, and the overall outcome of the disease may be tumor-type dependent. Here, we are the first to show that CXCL10 may serve as a negative prognostic factor in patients with NSCLC.

To gain more insight into the pleiotropic tendency of CXCL10, we looked at the overarching effect of IFNs-induced ISG response. Both type I and type II IFNs can elicit the transcription of ISGs, which include the IFN-inducible CXCL9/CXCL10/CXCL11 chemokines. In the current study, we investigated whether the “sum total” of circulating proteins can result in the activation of IFN/ISG response within myeloid cells via newly established sFIS assay [36]. In patients with stage I NSCLC treated with SBRT, increased IFN/ISG response activity was associated with a better PFS. This result emphasizes that although separate IFN-related proteins do not give a consistent result, the overall interferon-related inflammatory potential of the circulating proteins was increased after SBRT treatment in patients with stage I NSCLC. This was not the case for plasma samples from patients with stage III NSCLC treated with CCRT.

Given that ICD-related proteins, released by treatment-induced stressed or dying cells, act predominantly locally, we decided to perform a broader screening of the plasma proteome and the association thereof with PFS. In the current study, we identified CD244 as a potential prognostic biomarker in patients with stage I NSCLC. CD244 is a Signaling Lymphocyte Activation Molecule (SLAM) of the family of immunoregulatory transmembrane receptors, found on many immune cell types [52]. Recent studies have linked the CD244 inhibitory signaling to maintaining an exhausted phenotype in NK- and T-cells in chronic infection (i.e., SARS-CoV-2) and cancer, leading to tumor growth [53,54,55]. Our results are in line with the current literature, as increased circulating levels of CD244 at baseline tended to be associated with a worse PFS, probably due to a more immunosuppressive environment. To our knowledge, no clinical data are available regarding the potential immunosuppressive role of CD244 in patients with NSCLC. In previous studies, CD244 has already been shown to be co-expressed on CD8+ T-cells with other immunoregulatory receptors, including PD-1, CD160, CTLA-4, and TIM-3 in both mouse cancer models and patients with cancer [56,57,58,59]. Altogether, combined with our findings, it further strengthens the hypothesis that CD244 acts as an immunosuppressive receptor in patients with NSCLC. CD244 may therefore be a potential new therapeutic target to overcome resistance to existing ICIs.

Although we identified IL-10 and Oncostatin-M (OSM) as potential negative predictive biomarkers, the expression levels of both proteins did not alter during SBRT treatment. Interestingly, we found that the expression levels of OSM correlated with the COPD status of these patients, which is therefore considered as a non-significant finding due to bias. In support of this, previous studies have already shown that OSM plays an important role in a wide spectrum of inflammatory diseases, including COPD [60]. For IL-10, the Cox Proportional-Hazards model revealed that low circulating plasma levels were associated with a better PFS. Strikingly, the negative association with PFS already appeared to be present at baseline; the expression levels remained unaltered during treatment. IL-10 is an immunoregulatory cytokine that acts on many cells of the immune system where it has profound anti-inflammatory functions [61]. In line with our results, several studies have reported that increased IL-10 levels in the blood of patients with cancer may predict a worse outcome [62,63,64].

In patients with stage III NSCLC, we identified two immune-related proteins that were positively associated with PFS: CR2 and IFNGR2. CR2, also known as CD21, is a receptor for complement C3 and is expressed on the surface of B-cells, allowing the complement system to play a role in B-cell activation and maturation [65]. Although anti-tumor immunity is widely considered to be driven by T-cells, emerging evidence reveal that tumor-infiltrating B-cells (TIL-Bs) in NSCLC also correlate with patient prognosis [66]. Upon entering the local microenvironment, tumor antigens released from lung cancer cells aid in B-cell activation and trigger B-cell mediated antigen presentation [67]. These activated TIL-Bs can efficiently present antigens to CD4+ TILs and subsequently alter the CD4+ TIL phenotype. These activated TIL-Bs (CD19+/CD20+/CD69+/ CD27+/CD21+) have been shown to be associated with an effector T-cell response (IFNγ+/CD4+ TILs), whereas exhausted TIL-Bs (CD19+/CD20+/CD69+/CD27−/CD21−) have been shown to be associated with a regulatory T-cell (Treg) phenotype (FoxP3+/CD4+ TILs) [67]. In addition, high densities of activated TIL-Bs together with low densities of Tregs (FoxP3+/CD4+) in NSCLC tumors have consistently been associated with a better clinical outcome [67,68,69]. In line with the potential prognostic value of CR2, we here showed that increased circulating levels of CR2 are associated with a better PFS. Furthermore, CCRT seems to lower the circulating CR2 levels in both groups (low vs. high CR2 at baseline) towards the end of treatment, probably due to an overall immunosuppressive effect of chemotherapy, which can markedly reduce the number of lymphocytes. To the best of our knowledge, this is the first study reporting a positive association between circulating CR2 levels and PFS in patients with NSCLC. Aside from CR2, we showed that high baseline IFNGR2 levels may also have prognostic value. IFNGR2 forms together with IFNGR1 a heterodimer that is known as the human interferon-gamma (IFN-γ) receptor. Upon IFN-γ binding, the intracellular domains of IFNGR2 oligomerize and transphosphorylate IFNGR1, resulting in the activation of downstream signaling components that regulate gene expression through binding to gamma-activated site elements in the promotors of interferon-stimulated genes [70]. IFN-γ signaling coordinates several biological responses, primarily involved in host defense and immune surveillance, but also in the establishment of adaptive immunity [71]. For example, IFN-γ signaling has been shown to stimulate the polarization of macrophages towards the M1 pro-inflammatory phenotype [72,73], the upregulation of proteins of the antigen processing and presentation machinery and co-stimulatory molecules in antigen-presenting cells [74,75], and CD8+ T-cell proliferation following antigen exposure [76]. Upregulation of components of the IFN-γ signaling in the circulation may therefore be an indicator of an active immune system, which is required to kill cancer cells and to inhibit tumor growth through responses elicited by its innate and adaptive arms [77].

The results from this prospective study should be interpreted with some caution. First of all, as this study was set up as an exploratory study, only a small number of patients were included, hence the results cannot be accurately interpreted for the general population of patients with NSCLC and can lead to false negative results. A small study such as this is limited in its statistical power, therefore we considered the nominal *p*-value as a significance threshold rather than the multiple-testing adjusted *p*-value cut-off. Subsequent studies to validate these findings will require multiple testing adjustment. Secondly, a true baseline sample was missing in the group patients who were treated with CCRT. Within this group, the baseline blood sample was taken before the first fraction of RT and not specifically before the first cycle of chemotherapy. Therefore, the baseline expression levels of immune-related proteins may have already been affected by chemotherapy, which is an immunosuppressive agent. Still, the impact of radiotherapy could be validly looked at in this group. Third, long term treatment-induced changes could not be assessed as no blood samples were collected several weeks after RT. Furthermore, although it was not the scope of this exploratory study, a direct comparison of the levels of immune-related proteins in the TME and the levels of these proteins in the circulation would highly strengthen the data interpretation. In addition, in future studies, the expression levels of identified receptor-related proteins should be assessed on the surface of circulating immune cell types. Addressing these shortcomings in future prospective trials will ultimately result in a better understanding of the actual immune status of an individual patient before and during treatment. Currently, these clinical trials are already ongoing in patients with stage III and stage IV NSCLC (IPON-1 trial, ClinicalTrials.gov Identifier: NCT0443214 and Re-induction trial, ClinicalTrials.gov Identifier: NCT03406468, respectively). In both trials, peripheral blood mononuclear cells are isolated at pre-defined time-points, allowing to assess the phenotype of these circulating immune cells at baseline and during treatment.

When designing clinical studies focusing on the identification of blood-based immunological biomarkers in cancer patients treated with immunomodulatory agents and (chemo)radiotherapy, both results and limitations of this exploratory study should be considered. First of all, the prognostic and/or predictive value of the identified biomarkers should be prospectively analyzed in larger cohorts. Several immune-RT trials are already ongoing. For example, IMMUNOSABR is a randomized phase II trial combining SBRT with the interventional immunotherapeutic drug Darleukin (L19-IL2) in patients with stage IV NSCLC [78]. This trial includes an exploratory blood-based biomarker study on selected molecules that may be related to the treatment-induced immune response. Although important, this illustrates a recurring issue: translational biomarker research is often only included in interventional studies rather than in studies in which patients are only treated with standard of care. Given the immunomodulatory potential of radiotherapy and the increasing use of ICIs in both the curative and palliative setting, it is of utmost importance to identify blood-based immunological biomarkers that can be used (1) for monitoring the treatment response, (2) for the selection of patients that will most likely be responsive to ICIs, and (3) to define synergistic treatment combinations. In this regard, the results of our study provide a good starting point to implement blood-based immune profiling analyses in clinical trials.

## 5. Conclusions

In conclusion, we showed that the baseline expression levels of several circulating immunogenic proteins are correlated with PFS: in patients with stage I NSCLC, CD244 was identified as a potential negative prognostic biomarker. In patients with stage III NSCLC, CR2 and IFNGR2 were identified as potential positive prognostic biomarkers. Furthermore, treatment-induced changes of the expression levels of circulating immunogenic proteins identified CXCL10 and IL-10 as additional negative predictive biomarkers. As this clinical study was a prospective exploratory trial, these findings should be validated in larger patient cohorts.

## Figures and Tables

**Table 1:** Patient characteristics.

Patient Characteristics	General *n* = 44	Group 1—SBRT *n* = 26	Group 2—CCRT *n* = 18	*p*-Value
Gender (male, %)	27 (61.4%)	18 (69.2%)	9 (50.0%)	-
Age (years)	68.6 (±9.7)	73.0 (±7.8)	62.2 (±8.8)	<0.001
BMI (kg/m^2^)	26.5 (±4.3)	26.8 (±4.9)	26.1 (±3.5)	0.89
Active smoker (*n*, %)	13/43 (30.2%)	7/25 (28.0%)	6/18 (33.3%)	0.75
Pack years	39.2 (±16.4)	43.8 (±17.3)	32.7 (±13.0)	0.13
Diabetes (*n*, %)	7 (15.9%)	6 (23.1%)	1 (5.6%)	0.21
COPD (*n*, %)	15/35 (42.9%)	12/21 (57.1%)	3/14 (21.4%)	0.046
FeV1 (%)	74.5 (±23.4)	67.2 (±22.7)	85.8 (±20.2)	0.008
FVC (%)	102.8 (±20.1)	97.9 (±18.7)	110.2 (±20.5)	0.098
DLCO (%)	66.6 (±20.8)	59.8 (±20.4)	75.9 (±17.6)	0.034
RT dose (Gy)	55.0 (±7.7)	49.7 (±4.3)	63.2 (±3.1)	<0.001
Pathology				
Adenocarcinoma	21 (47.7%)	11 (42.3%)	10 (55.6%)	-
Squamous carcinoma	15 (34.1%)	10 (38.5%)	5 (27.8%)	-
Large cell carcinoma	1 (2.3%)	-	1 (5.6%)	-
NET	2 (4.5%)	1 (3.8%)	1 (5.6%)	-
Unspecified NSCLC	5 (11.4)	4 (15.4%)	1 (5.6%)	-
Progressive disease (*n*, %) PFS (months)	23 (52.3%) 20.2 (±12.7)	13 (50.0%) 23.8 (±10.7)	10 (55.6%) 15.0 (±12.8)	0.031-
Median PFS (months)	21.5 (±12.7)	25.0 (±11.5)	7.8 (±12.8)	0.031
Death (*n*, %)	21 (47.7%)	11 (42.3%)	10 (55.6%)	-
OS (months)	25.1 (±11.1)	27.9 (±7.6)	21.0 (±13.3)	0.12

BMI, body mass index; CCRT, concurrent chemotherapy and radiotherapy; COPD, chronic obstructive pulmonary disease; DLCO, diffusing capacity for carbon monoxide; FeV1, forced expiratory volume in 1 s; FVC, forced vital capacity; NET, neuroendocrine tumor; NSCLC, non-small cell lung cancer; OS, overall survival; PFS, progression-free survival; RT, radiotherapy; SBRT, stereotactic body radiotherapy.

## Data Availability

Data are contained within the supplementary material. The NPX data (Olink) presented in this study are available in the Supplementary Data file.

## Supplementary Materials

The following are available online at www.mdpi.com/article/10.3390/cancers13246259/s1, Figure S1. Differential circulating levels of immunogenic cell death-related proteins at base-line in patients with stage I and stage III NSCLC, Figure S2. Circulating levels of interferon-inducible chemokines and plasma-induced INF/ISG response activity in patients with stage III NSCLC treated with CCRT, Figure S3. Circulating levels of Oncostatin-M are associated with the COPD status of patients with stage I NSCLC, Supplementary Data provided separately attached as an Excel file.
