# Supplementary material for: Identification of Potential Prognostic and Predictive Immunological Biomarkers in Patients with Stage I and Stage III Non-Small Cell Lung Cancer (NSCLC): A Prospective Exploratory Study
Source: Cancers (Basel). 2021 Dec 13;13(24):6259. doi: 10.3390/cancers13246259 (PMC8699057; doi:10.3390/cancers13246259)
Supplement: Supplementary file 1 [file cancers-13-06259-s001.zip › cancers-1468832-supplementary/cancers-1468832-supplementary.pdf]

Supplementary Materials: Identification of Potential Prognostic and Predictive Immunological Biomarkers in Patients with Stage I and Stage III Non-Small Cell Lung Cancer (NSCLC): A Prospective Exploratory Study

Rianne D.W. Vaes, Kobe Reynders, Jenny Sprooten, Kathleen T. Nevola, Kasper M.A. Rouschop, Marc Vooijs, Abhishek D. Garg, Maarten Lambrecht, Lizza E.L. Hendriks, Marijana Rucevic and Dirk De Ruysscher

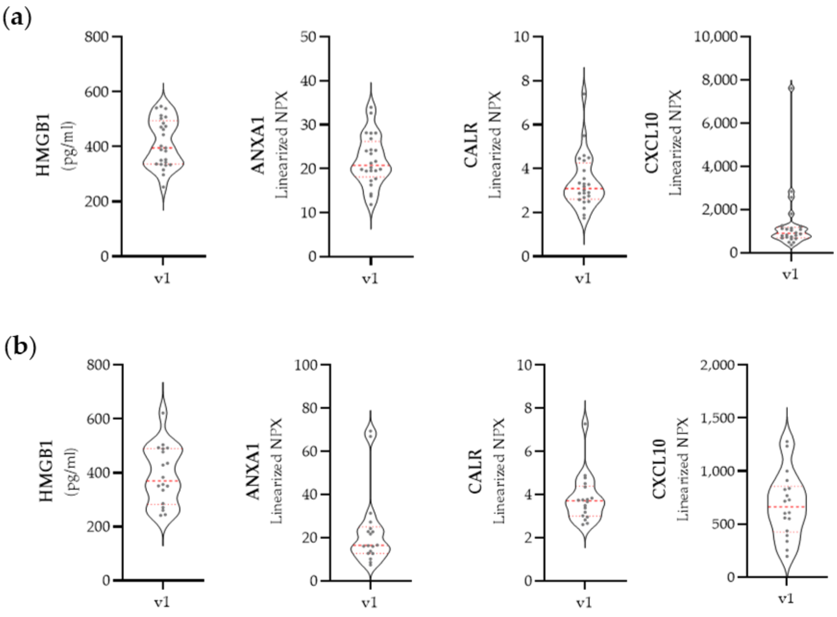

Figure S1. Differential circulating levels of immunogenic cell death-related proteins at baseline in patients with stage I and stage III NSCLC. Circulating levels of immunogenic cell death (ICD)-related proteins were assessed by ELISA (HMGB1) or by the Proximity Extension Assay from Olink (ANXA1, CALR, and CXCL10) in (**a**) patients with stage I NSCLC (n = 25) and (**b**) patients with stage III NSCLC (n = 18). The measured values are presented in a violin plot, showing the distribution of these values at baseline (v1). The median is indicated by the dashed red line, the quartiles are indicated by the rec dotted lines.

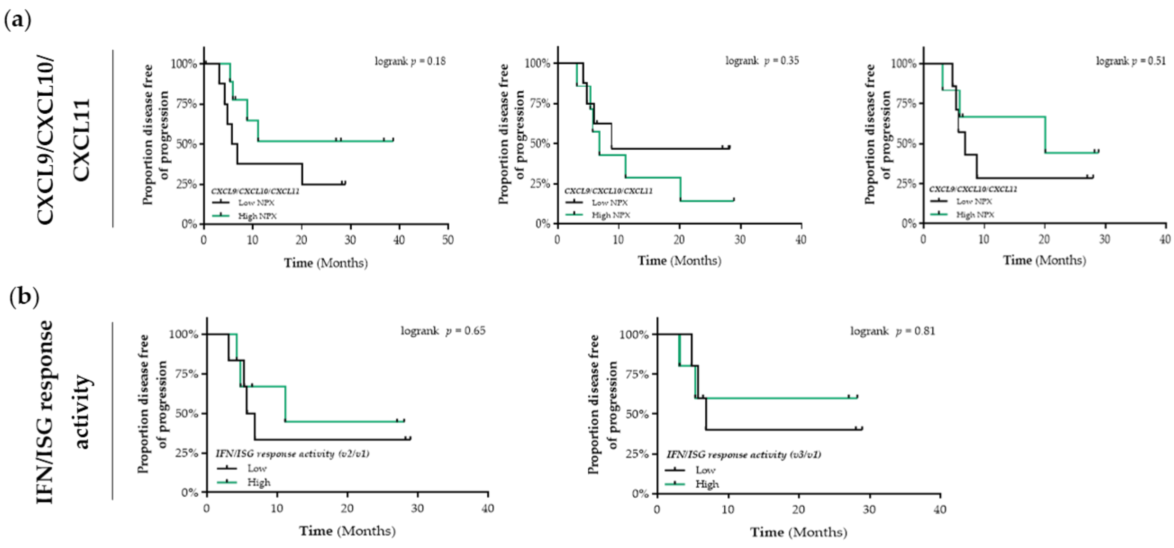

Figure S2. Circulating levels of interferon-inducible chemokines and plasma-induced INF/ISG response activity in patients with stage III NSCLC treated with CCRT. (**a**) Circulating levels of CXCL9, CXCL10, and CXCL11 in patients with stage III NSCLC (n = 18) at baseline (v1), after the 3rd fraction of RT (v2), and after the final fraction of RT (v3) were assessed by the Proximity Extension Assay from OLINK. Patients were stratified into low (black lines) or high (green lines) expression-based "risk-groups" by considering the median NPX value of the mean NPX (CXCL9, CXCL10, and CXCL11) as a cut-off, followed by Kaplan-Meier plotting on the patient's PFS. (**b**) Plasma-stimulated activation of interferon-stimulated response elements (ISRE) coding for interferon (IFN)-stimulated genes (ISG) was assessed by measuring luciferse activity in media from THP1-Dual™ reporter cells that have been treated for 24 h with plasma samples. IFN/ISG response activity values were normalized against the IFN/ISG activity measured in media from THP1-Dual™ reporter cells treated for 24 h with pre-treatment (v1) samples of each individual patient. Patients were stratified into low (black lines) or high (green lines) IFN/ISG response activity-based "risk-groups" by considering the median IFN/ISG activity value as a cut-off, followed by Kaplan-Meier plotting on the patient's PFS. In all graphs, respective logrank test p-values are displayed. A nominal p-value less than 0.05 was considered to be statistically significant and is indicated through an asterisk (*).

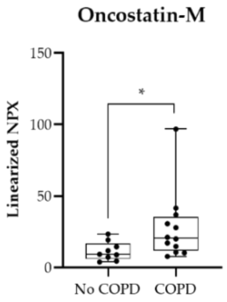

Figure S3. Circulating levels of Oncostatin-M are associated with the COPD status of patients with stage I NSCLC. Circulating levels of Oncostatin-M (OSM) in patients with stage I NSCLC (n = 21) at baseline (v1) are associated with the COPD status of these patients. Patients with COPD have significantly increased circulating levels of OSM compared to patients without COPD (28.0 ± 24.2 vs. 11.5 ± 6.6, p = 0.01).

The Supplementary Data is provided separately attached as an Excel file.
